# European Laryngological Society consensus statement on optimal monitoring schedules after treatment for early glottic cancer: a risk-stratification

**DOI:** 10.1007/s00405-025-09609-0

**Published:** 2025-08-04

**Authors:** Malgorzata Wierzbicka, Agatha Baidun, Andy Bertolin, Marco Lionello, Giovanni Succo, Berit Verbist, Davide Farina, Martine Hendriksma, Marc Remacle, Ricard Simo, Elisabeth Sjogren, Cesare Piazza

**Affiliations:** 1 Faculty of Medicine, Wroclaw University of Science and Technology, 27 Wybrzeże Stanisława Wyspiańskiego st, Wrocław, 50-370 Poland; 2 Institute of Human Genetics Polish Academy of Sciences, 32 Strzeszyńska st., Poznań, 60-479 Poland; 3 Department of Otorhinolaryngology and Head and Neck Surgery, Leiden University Medical Center, Albinusdreef 2, Leiden, 2300RC the Netherlands; 4 Unit of Otorhinolaryngology – ENT Dept, Vittorio Veneto Hospital, Vittorio Veneto, Italy; 5 Department of Oncology, University of Turin, San Giovanni Bosco Hospital, Turin, Italy; 6 Department of Radiology, Leiden University Medical Center, Leiden, the Netherlands; 7 Department of Radiological Sciences (DSMC), University of Brescia, Brescia, Italy; 8 Department of Otolaryngology, Centre Hospitalier du Luxembourg, Luxembourg, Luxembourg; 9 Department of Otorhinolaryngology Head and Neck Surgery, Guy’s and St, Thomas’ Hospital NHS Foundation Trust, London, UK; 10 Unit of Otorhinolaryngology – Head and Neck Surgery, ASST Spedali Civili of Brescia, Brescia, Italy; 11 Department of Surgical and Medical Specialties, Radiological Sciences, and Public Health, School of Medicine, University of Brescia, Brescia, Italy

**Keywords:** Follow-up, Laryngeal cancer, Early glottic carcinoma, T1, T2, Recurrence

## Abstract

Early glottic cancer has an excellent prognosis provided that recurrences are detected in a timely manner. However, current guidelines lack specific recommendations for intervals or interventions during follow-up, and primarily advocate surveillance in the most advanced stages where the benefits are actually the lowest. This consensus statement introduces a risk-stratification-guided follow-up schedule for T1-T2N0 patients, aiming to optimize oncologic and functional outcomes while ensuring early detection of residual or recurrent disease to preserve organ function. Separate protocols are outlined for surgical and non-surgical patients, including endoscopic examination, radiological imaging, and thyroid function screening. Also, the pathway to reach this routine observation phase is described, specifying the criteria and timing of a second-look microlaryngoscopy.

## Background

The larynx, despite its small size, is a complex structure in which primary tumors can exhibit heterogeneous extensions and behaviors. Prognosis varies significantly, ranging from a 99% cure rate for early glottic cancers, to only a few percent for advanced supraglottic lesions with hypopharyngeal extension [1, 2].

A key aspect of laryngeal cancer management is the high potential for organ preservation in timely detection of limited recurrences that can still be treated with salvage surgery or (chemo-)radiotherapy ([C]RT) [3–10]. Many existing guidelines, such as those of the National Comprehensive Cancer Network (NCCN), base their follow-up protocols primarily on prognosis derived from the clinical/pathological TNM staging as well as primary location and pathohistological risk factors of the primary tumor [11]. However, using these “classical” parameters to estimate risk of recurrence leads to higher surveillance of the most advanced stages, where the benefits are actually the lowest, as effective larynx sparing or even curative treatment options are often no longer available [12].

Additionally, follow-up is often recommended for at least 5 years after treatment [11] but the method of surveillance is usually only described in general terms, without specific recommendations for intervals or interventions. And although numerous oncological studies have investigated follow-up intensity in different cancers [13–18], findings remain contradictory, as some studies show a link between a close follow-up program and early diagnosis of recurrences, while others do not [19, 20]. This is true even in limited glottic lesions. In a Finnish study of early glottic cancer, 68% of local recurrences were diagnosed during routine follow-up versus 45% presenting with new symptoms [21]. Conversely, a Norwegian study reported that most recurrences were symptomatic, and symptom-based detection correlated with improved survival after salvage [22]. The authors suggested that patient self-detection of ‘red flag’ symptoms and self-initiated visits may be underappreciated prognostic factors, potentially optimizing follow-up strategies.

As the benefits of rigorous follow-up are still unclear, it is important to consider that it also has the disadvantage of causing unnecessary discomfort and anxiety to patients and of creating significant costs for healthcare systems. This is supported by recent studies on cost-benefit ratios in follow-up, advocating for the reallocation of healthcare resources by reducing ineffective check-ups and instead implementing multi-specialty care for the patients who will truly benefit [23, 24]. Moreover, not all patients are fully adherent to the offered surveillance, with factors such as age (≥ 70 years), unmarried status, higher education level and a negative history of surgery for premalignant or malignant lesions associated with lower follow-up attendance [25]. Therefore, it follows that an innovative approach to laryngeal cancer follow-up should emphasize monitoring patients who can be offered effective salvage, and not necessarily the ones with the highest risk of recurrence. This principle applies especially for patients with T1-T2N0 glottic cancer as it has a good to excellent prognosis regarding recurrence-free survival, organ preservation rates and laryngeal function [26, 27]. In these patients, risk-stratification-guided surveillance translates to opportunity-stratification-guided scheduling.

Based on this premise, we hypothesize that in T1-T2N0 glottic cancers, high organ preservation and cure rates remain achievable when residual tumors are diagnosed without undue delay and/or recurrences are detected in a timely manner. Therefore, follow-up protocols must differ from those of other primary locations and stages. Despite the low recurrence rates, an intensive follow-up schedule remains justified. This publication aims to discuss the factors involved in risk-stratification-guided surveillance for T1-T2 glottic cancers and to propose optimal post-treatment monitoring schedules.

## Methodology

The guidelines were developed through a structured, consensus-driven process involving a multidisciplinary panel of experts. Initial drafts were constructed following a comprehensive review of the current literature and existing clinical practice standards. The panel convened for a series of structured meetings to discuss, refine, and prioritize key recommendations. Given the interdisciplinary nature of the group and the complexity of the topic, a formal Delphi process was not used. Instead, consensus was reached through open discussion, iterative feedback, and collective agreement during the meetings. Disagreements were resolved through additional dialogue until a consensus was achieved. All recommendations were reviewed and approved by the entire panel prior to finalization of the guidelines.

## Surgical versus non-surgical treatment

The primary treatment modalities for T1-T2 glottic carcinoma are transoral laser microsurgery (TOLMS) and RT. Additionally, open partial laryngectomies (OPLs), including both horizontal and vertical approaches, are viable options for a subset of patients and both TOLMS and horizontal OPL procedures are nowadays performed according to standardized and codified surgical techniques [28, 29]. All these therapeutic approaches provide high levels of local control, as well as laryngectomy-free and disease-specific survival rates [28–31], though their functional outcome profiles may vary slightly. Individual treatment choices are tailored to the specific patient profile, institutional conditions, and patient preferences. However, as treatment selection falls outside the scope of this consensus statement, it will not be discussed further. Regardless of the treatment modality, the risk groups for failure remain the same, as each alternative treatment must achieve comparably effective outcomes. In practice, despite similar oncologic outcomes, post-treatment monitoring strategies can differ significantly between surgical (TOLMS/OPL) and (C)RT patients with respect to the frequency of follow-up visits and the methods of examination. Additionally, the use of imaging such as computed tomography (CT) and magnetic resonance imaging (MRI) varies between treatment groups, with surgical and non-surgical healthcare professionals possibly applying different indications for imaging when screening for recurrence and assessing post-treatment changes.

## After treatment: establishing the clinical scenario

Following primary treatment with curative intent, an established schedule of monitoring visits should be implemented, regardless of whether the treatment was surgical or non-surgical. However, even with adequate therapy, uncertainties may arise regarding the effectiveness of RT or the completeness of surgical resection. Before concluding the treatment phase and transitioning the patient to routine observation, a thorough assessment should be conducted, ensuring the patient has reached an optimal post-treatment state. Three clinical scenarios can be defined:

1. adequate treatment (radical resection or complete response to RT): the patient proceeds to routine follow-up according to the appropriate schedule.
2. inadequate treatment (residual disease): the patient requires further treatment, such as revision TOLMS, (C)RT, OPL, or total laryngectomy (TL), depending on the clinical scenario.
3. unclear treatment status: if the effectiveness of treatment remains uncertain, additional evaluation is necessary before transitioning to standard surveillance.

If treatment status remains unclear at the end of therapy, and the therapeutic phase cannot be definitively concluded, the patient should not be referred for scheduled observation but should instead undergo further reassessment. However, the approach for evaluating this initial treatment response differs between RT and surgical treatment. As RT lacks additional information on surgical margins, treatment response must be evaluated using flexible endoscopy in the first three months after treatment completion. Inadequate healing should raise suspicion of an incomplete response, and further diagnostics, such as imaging and second-look microlaryngoscopy (SL MLS), should be considered. On the other hand, in surgical cases, resection margins play a crucial role and offer immediate guidance for further management and decision-making regarding SL MLS, although margin assessment after TOLMS has its unique challenges.

## Role of margins

When surgical margins are clearly negative or positive following TOLMS, subsequent management is well defined. While those with negative margins can proceed to routine follow-up, patients with positive margins require additional treatment such as revision TOLMS, OPL, TL, or (C)RT. However, difficulties arise when margin status is ambiguous. Unclear, close, or non-evaluable resection margins may result from several factors including shrinkage of small specimens, carbonization of edges, and inclusion of margins in frozen section analysis [24, 32–36]. Margin assessment in T1-T2 glottic cancers can also be complicated by the surgical technique, particularly when using a piecemeal rather than en-bloc excision approach, which is sometimes necessary for bulky tumors with complex three-dimensional orientations.

Positive margins following TOLMS have been reported in 10–50% of cases [32, 34], with higher rates observed in non-academic centers and institutions with lower caseloads [37]. However, the prognostic significance of positive margins remains debated. While some studies associate positive margins with increased recurrence risk [38–40], others report no significant impact [36, 41, 42]. For instance, Ansarin et al. demonstrated increased recurrence risk in patients with close or positive margins who did not receive further treatment, compared to those with negative margins [38]. Charbonnier et al. found significantly reduced 5-year disease-free survival in patients with positive margins (*p* = 0.009) and those with deep involvement of the vocal muscle (*p* = 0.004) [40]. Conversely, Michel et al. found no significant impact on the carcinologic course, reporting a 5-year recurrence-free survival of 91.7% in patients with negative margins versus 95% in those with positive margins [42]. Likewise, Sigston et al. observed no significant difference in recurrence rates when comparing histologically clear with suspicious margins [36]. These mixed findings highlight the critical role of the subjective assessment of resection radicality by the surgeon. Studies suggest that the surgeon’s intraoperative evaluation remains one of the most important factors in determining the completeness of resection [43, 44]. The value of this clinical insight, particularly by experienced endoscopists, should therefore not be underestimated when interpreting margin status in cases of uncertainty. To enhance margin assessment, standardized techniques for specimen orientation and fixation during TOLMS have been developed. These approaches facilitate reliable deep margin evaluation, aiding decisions on SL MLS or determining follow-up intensity [45].

The evaluation of surgical margins after OPL is usually more straightforward given the open-neck approach applied in such procedures, although a distinction should be made between the results of intraoperative frozen sections and the final pathological examination. As an organ-sparing surgery, OPL necessarily needs intraoperative frozen sections in order to certify the oncological radicality. Using the standardized approaches of the open partial horizontal laryngectomies (OPHLs), critical areas in need of assessment are the anterior commissure (AC) in case of OPHL Type I, suprahyoid epiglottis in case of OPHL Type IIa, lateral crico-arytenoid muscle and crico-arytenoid joint in case of OPHL Type II with removal of one arytenoid, and residual cricoid cartilage and subglottis in case of OPHL Type III. When positive, the resection has to be expanded until obtaining negative margins, passing from OPHL Type I to Type IIa, or from Type IIa to Type IIb, or from Type II to Type III. Thereafter, the margins of the surgical specimen have always to be checked again upon definitive pathology, which is generally much more predictable than after TOLMS. This possibility of shifting the OPHL type, based on the status of the frozen sections, is one of the advantages of this procedure [46].

On the other hand, intraoperative examination with frozen sections is characterized by a certain imprecision degree [47]. This is especially true when considering salvage OPL after failed organ preservation protocols [48]. Therefore, the status of the margins at the definitive examination is the parameter that really influences the patients’ prognosis. With a few exceptions [49], the literature on the topic agrees in considering positive resection margins as an unfavorable prognostic factor and an indication for adjuvant treatment [50–55]. Although the larynx “tolerates” minimal resection margins, this can potentially expose patients to a greater number of loco-regional relapses [50, 51]. The authors consider intraoperative frozen sections essential in OPL, as well as the availability of a team of expert laryngeal pathologists. The true positive margin is the one confirmed by the definitive examination, both on the surgical specimen and on the resection enlargements. OPL patients with positive surgical margins have to be discussed at a multidisciplinary tumor board for adjuvant treatment.

## Second-look microlaryngoscopy

Regardless of the primary treatment method applied, a second-look procedure may be warranted. As discussed above, evaluating surgical margins, especially after TOLMS, can be challenging, making SL MLS an essential tool in these scenarios. For patients treated with (C)RT, information on the surgical margins is missing and more invasive diagnostics like SL MLS may be necessary at an earlier stage. However, recommendations regarding whether the patient should undergo SL MLS remain inconsistent, despite a meta-analysis by Verro et al. providing pertinent data on the utility of SL MLS following TOLMS [56], and the topic has also been debated in the study by Shenoy et al. [57].

The European Laryngological Society (ELS) incorporated this concept into their 2014 follow-up recommendations for laryngeal cancer [58], stating that SL MLS is mandatory for positive margins and recommended for close or non-evaluable margins. Since carbonization can make it difficult to distinguish between positive and unclear margins, some authors group these cases together and recommend SL MLS or close follow-up based on the surgeon’s preference [24, 34]. Others advocate adapting the approach based on the depth and number of positive margins, reasoning that a watch-and-wait policy may be reasonable for a single superficial or close margin, depending on the surgeon’s clinical judgment [34, 41], but that multifocal superficial and deep positive margins should be surgically verified via SL MLS [7, 59, 60]. The optimal timing of SL MLS remains controversial. Some authors recommend an early second-look procedure at 3–4 weeks after surgery [41, 61], while others suggest waiting up to 12 weeks [33, 62]. The ELS provides no specific guidance, instead proposing a broad range of 1–8 months [58]. Beyond oncologic surveillance, SL MLS offers additional benefits, such as the management of laryngeal webs, synechiae, or granulomas.

Based on available literature and meta-analyses [56], SL MLS, with or without additional resection, is indicated in the following scenarios: (1) positive deep margin(s), (2) more than one positive superficial margin, (3) uncertain margin status requiring further evaluation, and (4) healing difficulties post-treatment. SL MLS is particularly recommended for high-risk cases, including tumors involving the AC, deep vocal fold muscle, or paraglottic space (PGS), where recurrence rates are higher. If SL MLS is deemed necessary, it should be scheduled at least 3 months post-treatment. Initial healing and re-epithelialization typically occur within 12 weeks, making earlier assessments premature and potentially leading to overtreatment. The optimal SL MLS timing allows for an accurate evaluation of the glottis based on prior surgical knowledge and the actual post-treatment condition of the laryngeal inlet, assessed using white light (WL) and narrow-band imaging (NBI) whenever possible.

## The follow-up phase

Once adequate primary treatment has been established, patients transition to routine follow-up. Primary goals of follow-up include early detection of local or loco-regional recurrence, identification of distant metastases or metachronous tumors, assessment of functional outcomes and quality of life, detection and management of complications or late sequelae, rehabilitation, smoking cessation, and patient education [58]. However, detailed considerations regarding follow-up may differ depending on the tumor location and treatment modality used. In general, supraglottic and subglottic regions have a more extensive lymphatic network, making them more prone to regional lymph node metastasis compared to tumors confined to the glottic plane in early stages [63]. Consequently, follow-up for these cases must include careful evaluation of lateral cervical lymph nodes. Subglottic lesions in particular show a higher tendency to spread along the paratracheal nodes, necessitating focused monitoring in these areas [64]. However, T1-T2N0 glottic carcinomas rarely metastasize to the neck and the focus is on local control. Here, the mode of treatment is the primary distinguishing factor and treatment-specific challenges need to be considered. Regardless of modality, follow-up visits must include transnasal videolaryngoscopy with both WL and vascular magnification filters, such as NBI, to enhance lesion detection [65]. Image and video documentation of endoscopic findings are crucial for longitudinal comparisons during follow-up.

### Follow-up after radiotherapy

Post-RT endoscopic examinations face challenges due to radiation-induced changes, such as mucosal thickening and inflammation, which reduce the resolution of standard imaging techniques [66]. While tumor-related mucosal alterations typically subside around 3 months after RT, NBI remains of limited utility due to poor visibility of microvascular patterns through the thickened, radiation-altered mucosa. Studies indicate that new longitudinal vasculature continues developing for up to 9 months post-RT, further complicating early recurrence detection [67]. As a result, endoscopic assessment with WL remains challenging, and additional surveillance strategies such as imaging should be considered in many post-RT scenarios.

### Follow-up after surgery

Endoscopic evaluation is generally easier after surgery than after RT due to less chronic edema and a smoother epithelium. Additionally, intraoperative removal of false vocal folds can significantly enhance visualization of critical areas, including the ventricle, subglottis, and epiglottic petiole. This facilitates early identification of contour changes that could indicate recurrence. Furthermore, there is information from the margins to help guide follow-up. Studies have confirmed that integrating NBI with WL imaging improves endoscopic assessment following TOLMS demonstrated by Witkiewicz et al. [68] and corroborated by Lukes et al. [69], who reported high sensitivity, specificity, and negative predictive value for NBI in detecting residual disease. Despite these advantages, laryngoscopy alone may not be sufficient for early identification of deep-seated, submucosal recurrences, which are more common after surgery than after RT, particularly after extended or repeated TOLMS resections. In such cases, targeted imaging, such as contrast-enhanced MRI or CT scans, may be warranted even in routine screening.

## Role of imaging

Although there is broad consensus on the potential role of diagnostic and functional imaging techniques for the surveillance of glottic cancer, indications and timing of scans are debated in the literature. On one hand, NCCN suggests limiting routine imaging surveillance to head and neck tumors locally advanced at diagnosis or those in areas that are difficult to assess on clinical examination. At the other extreme of the spectrum, the Neck Imaging Reporting and Data System (NI-RADS) recommends periodic scanning in all patients, starting 8–12 weeks after treatment completion, with follow-up scans scheduled every 6 months in the first year and every 8–12 months thereafter [70].

Targeted recommendations for patients treated for early glottic cancer are not available. Nonetheless, two factors support the use of CT and MRI, at least in T2 category: as abovementioned, the RT-induced mucosal changes hinder clinical evaluation in the short and mid-term, whereas after TOLMS, recurrences are often deeply seated in the submucosa, making them difficult to detect through clinical examination alone [9, 71]. Furthermore, Marchi et al. showed that adding imaging to endoscopic follow-up in high-risk patients treated with TOLMS for T2-T3 glottic cancer increased the detection rate of submucosal recurrences and the organ preservation rate [72].

The choice between CT and MRI is often dictated by practical considerations; easy access at relatively low cost and fast acquisition makes CT the workhorse in most centres. In recent years, CT has been boosted by the introduction of spectral techniques (such as dual-energy and photon counting CT). These advances promise improved contrast resolution, potentially allowing more accurate discrimination between different tissues and cartilage invasion [73–75]. MRI is more technically demanding and requires longer acquisition times, resulting in higher incidence of motion artefacts. Radial-k-space filling sequences are available on nearly all scanners and compensate for respiratory artefacts and mild movements. In addition, state-of-the-art scanners implement deep learning algorithms that can be applied across various sequences and are trained to improve the signal-to-noise ratio (SNR). This allows for either shorter acquisition time or reduced image noise, ultimately improving the overall quality. Finally, SNR is also significantly improved by scanning the larynx with surface coils, directly positioned on the anterior neck of the patient [76, 77]. However, it must be emphasized that the field of view with such coils is restricted, thus the acquisition protocol should always include at least one sequence obtained with the neck coil, to cover all nodal levels. Such a coil set-up requires careful and precise positioning of the surface coils, customized to individual anatomy and performed by an experienced technician.

The interpretation of imaging findings can be challenging with both CT and MRI. Hermans et al. proposed a 3-tiered classification of post-RT CT findings in patients treated for laryngeal or hypopharyngeal squamous cell carcinoma (SCC) [78]. Such classification showed good correlation with local control during a 2-year follow-up term, and allowed on average a 5.5-month earlier diagnosis than clinical examination. Nonetheless, the applicability of such CT criteria to post-surgical findings needs to be validated, and the diagnostic advantage over clinical examination should be confirmed in light of recent advances in endoscopic techniques. Similar to CT, King et al. proposed a stratification of MRI T2 findings based on signal intensity and morphology, which was applied to head and neck cancer (HNC) patients (non-specifically to laryngeal SCC) treated with CRT [79]. In addition, another 3-tiered scoring system was applied by Ravanelli et al. for patients scanned with MRI and surface coils during the follow-up of laryngeal cancer treated with TOLMS; in this classification, findings obtained with diffusion-weighted sequences were also integrated to differentiate recurrence (typically nodular in shape and characterized by an intermediate T2 signal and diffusion restriction) from post-surgical scar (low T2 signal without diffusion restriction) or edema/granulation (causing high T2 signal without diffusion restriction) both in the soft tissue as well as in the cartilages [71].

Conveying the information obtained with CT or MRI in a reliable and clinically useful radiological report requires standardized terms, to optimize the interdisciplinary communication. With such aim, the NI-RADS initiative proposes a classification of imaging findings at the primary site and in the neck observed in follow-up scans of patients treated for HNC [70]. Four classes are suggested, namely ‘no evidence of recurrence’ (NI-RADS 1), ‘low suspicion’ (NI-RADS 2), ‘high suspicion’ (NI-RADS 3), and ‘definitive recurrence’ (NI-RADS 4). For each class, advice on further management is also suggested; in particular, closer follow-up with imaging is suggested for NI-RADS 2 and a biopsy for NI-RADS 3. Such an approach makes NI-RADS an actionable tool, ideal for multidisciplinary case discussion. Interestingly, all the abovementioned classifications of imaging findings mirror the clinical scenarios previously described, emphasizing the importance of implementing such semantic in the multidisciplinary discussion, particularly in more complex cases.

Lymphatic spread of early glottic cancers is rare. In a systematic review, Sanabria et al. reported a pooled incidence for occult metastasis of 8% (95% CI, 2.7–13.3, I^2^ = 81%) [80]. Lymph node levels II and III are most frequently involved [80]. Pretreatment neck ultrasound with fine needle aspiration represents the most accessible and accurate test for evaluating lymph node status. Follow-up of the neck is done by clinical observation [11]. The role of positron emission tomography (PET)-CT in the follow-up after OPL is debated, given its low specificity. However, it could be considered in cases with local or loco-regional suspicious CT/MRI findings (in particular after adjuvant CRT), and for monitoring distant metastases or detecting second primaries.

## Post-treatment evaluation in T1-T2N0 carcinoma: moving towards a checklist

The aim of this consensus statement, additional to reviewing and discussing the factors involved in risk-stratification-guided surveillance, was to propose a post-treatment monitoring schedule for T1-T2N0 patients based on the general goals of follow-up and surveillance. These include maximizing long-term oncologic and functional outcomes, improving quality of life, and minimizing unnecessary or low-value care, taking into account that high organ preservation rates remain achievable in these patients when residual tumors are diagnosed without undue delay and recurrences are detected in a timely manner. A basic risk classification is provided in Table 1. Factors warranting an upgrade into a higher risk-group for T1-T2N0 were deemed to be local anatomy i.e., tumor localization in high-risk areas such as the AC or the posterior PGS, and positive lymph nodes [81, 82]. Table 2 shows the proposed follow-up schedule based on the risk classification as well as literature and expert consensus of the authors.

**Table 1:** Categorization of tumors according to the risk of recurrence and the possibility of applying an effective salvage treatment

Primary location	*Group I* Very low risk of failure after first-line treatment	*Group II* Moderate risk of failure after first-line treatment, effective salvage treatment if needed associated with improved survival	*Group III* High risk of failure after first-line treatment, still possibility of effective salvage treatment associated with improved survival
Larynx, glottis T1 surgery	+		
Larynx, glottis T1 radiotherapy	+		
Larynx, glottis T2 surgery		+	↑+ ^1,2^
Larynx, glottis T2 radiotherapy		+	↑+ ^1,2^

Legend: ↑ upgraded to the higher risk group due to adverse prognostic factors based on: ^1^ anatomy: anterior commissure involvement or posterior paraglottic space involvement or ^2^ N + stage

Group IV: Moderate or high risk of failure, not eligible for treatment of recurrence with established salvage methods. Redundant in T1-T2 glottis

**Table 2:** Proposed follow-up schedule according to risk category

Follow-up schedule (months)				3	6	9	12	16	20	24	28	30	32	36	42	48	54	60
Group	Risk of recurrence	Salvage feasible	Organ preservation feasible															
Group I all modalities	very low	yes	yes	1	1	1	1,3,4*	1	1	1,3,4*	1	1	1	1,3,4*	1	1,3,4*	1	1,3,4*
Group II surgery	moderate	yes	often	1	1	1	1,3	1	1	1,3	1	1	1	1,3	1	1,3	1	1,3
Group II radiotherapy	moderate	yes	in selected cases	1	1	1	1,3,4	1	1	1,3,4	1	1	1	1,3,4	1	1,3,4	1	1,3,4
Group III surgery	high	yes	in selected cases	1	1,2	1	1,2,3	1	1	1,2,3	1	1	1	1,3	1	1,3	1	1,3
Group III radiotherapy	high	yes	in selected cases	1	1,2	1	1,2,3,4	1	1	1,2,3,4	1	1	1	1,3,4	1	1,3,4	1	1,3,4

Legend: (1) routine head and neck examination (including videolaryngoscopy with white light and narrow-band imaging); (2) computed tomography (CT)/magnetic resonance imaging (MRI) larynx and neck; (3) chest imaging in high-risk patients (age and smoking); (4) blood tests (thyroid); 4* blood tests for radiotherapy group only

One of the proposed follow-up modalities in this schedule is chest CT. Pulmonary metastases are not expected in patients with early glottic cancer. However, due to shared risk factors such as advanced age and smoking history, patients with laryngeal cancer face an increased risk of developing second primary lung tumors, with reported incidence rates ranging from 4 to 10% [83–88]. To the best of our knowledge, there is no recent literature proposing specific recommendations regarding chest imaging during follow-up in patients with laryngeal cancer. Several studies have explored this topic in broader HNC cohorts, but their recommendations vary from screening all HNC patients [23, 89], to only those with higher-stage disease [90], or older age and excessive tobacco use [91]. In the absence of clear guidelines for HNC, it may be informative to consider more general guidelines for lung cancer surveillance. The NCCN guidelines recommend annual low-dose CT chest screening for all individuals aged 50 years or older with significant smoking history [92]. Similarly, the recently updated guidelines of the United States Preventive Services Task Force (USPSTF) recommend annual low-dose CT chest screening for those aged 50 to 80 years with a significant smoking history, though with slightly different pack-year criteria [93]. This strategy has been shown to reduce lung cancer related mortality by 20% and overall mortality by 6.7% through early detection [94]. Piersiała et al. studied the implementation of the USPSTF guidelines in laryngeal cancer patients and found suspicious pulmonary nodules in 52% of those meeting the screening criteria, with 6% confirmed to have metachronous lung malignancy. While most cases were detected in the first and second year after treatment, a notable number emerged up to 12 years later, indicating the importance of annual follow-up [95, 96]. However, the study did not report any outcomes on potential survival benefit. Based on the reviewed literature, this guideline recommends annual chest imaging in high-risk patients as long as they are in follow-up.

Another aspect requiring special attention in the follow-up, specifically after RT, is the screening for hypothyroidism. This is a known and fairly common complication after RT in HNC patients, with an estimated incidence of 36% according to a recent systematic review [97]. Interestingly, studies relying on clinical symptoms reported lower rates compared to those incorporating more extended follow-up [97, 98]. Others have found that most cases of hypothyroidism are subclinical [99, 100], emphasizing the limitations of symptom-based detection. Mulholland et al. investigated the optimal timing for detecting RT-related hypothyroidism in irradiated laryngeal cancer patients, finding that 42% developed the condition, with the majority diagnosed at or after 12 months [101]. Similar detection intervals have been reported in other studies [98, 99, 102]. Moreover, the thyroid function seemed to worsen over time, with 28% of the cases eventually meeting the threshold for thyroid hormone replacement therapy to prevent associated complications such as cardiovascular disease, obesity, and hypertension [103]. Based on these findings, authors recommend yearly screening of the thyroid function starting at 12 months after treatment.

As previously stated, lymphatic spread to cervical lymph nodes is rare in early glottic cancer and the follow-up of the neck is primarily done by clinical observation [11]. Yet, when imaging is required, there is no consensus in the literature on the optimal modality. A study on patients with orofacial malignancies and clinically suspect lymph nodes metastasis found that CT was more sensitive and specific than ultrasound [104]. A slight superiority of CT over ultrasound is also found in clinically N0 patients by Liao et al. [105]. Contrary, multiple studies report ultrasound as the preferred modality [106, 107] and others find no differences between the two [108, 109]. It should be noted that these studies were conducted during the diagnostic phase, and post-radiation changes in tissues may impact the accuracy of these imaging techniques. Based on this, the authors recommend screening the neck in higher-risk cases, using the same imaging (CT/MRI) recommended for local follow-up.

Finally, although the recommendations in this review do not extend to voice and swallowing rehabilitation, it is of course paramount that these functional parameters are carefully assessed and optimized throughout the patient’s entire residual life. As a matter of fact, especially in the older patient, late worsening dysphagia may hamper their swallowing capability, putting the laryngeal cancer survivor at higher risk for ab ingestis pneumonia than the standard elderly population. Given the broad scope and complexity of functional outcomes, we believe it would be more appropriate to establish a separate consensus statement that offers targeted, evidence-based recommendations for the follow-up of these parameters.

## Conclusions

Establishing a standardized follow-up strategy for early glottic cancer treated with laryngeal preservation surgical approaches or RT benefits both patients and healthcare providers. A structured approach enhances surveillance, facilitates early detection, and serves as a reference framework for individualized post-treatment management. Based on current evidence, this ELS consensus proposes a follow-up schedule for T1-T2 glottic carcinoma, including criteria and timing for SL MLS. The goal is to maximize salvage success rates while balancing healthcare resource allocation and cost-effectiveness.
